# Protein Profile Changes in Circulating Placental Extracellular Vesicles in Term and Preterm Births: A Longitudinal Study

**DOI:** 10.1210/endocr/bqaa009

**Published:** 2020-01-29

**Authors:** Ramkumar Menon, Chirantan Debnath, Andrew Lai, Dominic Guanzon, Shinjini Bhatnagar, Pallavi Kshetrapal, Samantha Sheller-Miller, Carlos Salomon

**Affiliations:** 1 Division of Maternal-Fetal Medicine & Perinatal Research, Department of Obstetrics & Gynecology, The University of Texas Medical Branch at Galveston, Galveston, Texas; 2 Translational Health Science and Technology Institute of India, Faridabad, Haryana, India; 3 Exosome Biology Laboratory, Centre for Clinical Diagnostics, University of Queensland Centre for Clinical Research, Royal Brisbane and Women’s Hospital, The University of Queensland, Brisbane, Australia; 4 Department of Clinical Biochemistry and Immunology, Faculty of Pharmacy, University of Concepción, Concepción, Chile

**Keywords:** preterm birth, exosomes, maternal plasma, fetal exosomes, biomarker

## Abstract

Spontaneous preterm birth (PTB) is a major obstetrical problem around the globe and the mechanisms leading to PTB are unclear. Recently, changes in the circulating levels of placental extracellular vesicles (EVs) during pregnancy have been associated with various pregnancy complications. However, progress in the field is hindered by the inability to isolate placental EVs from the maternal circulation. A longitudinal study design was used to determine the protein cargo present in circulating placental EVs in maternal plasma of term and PTB across gestation (ie, first, second, and third trimester). Placental-derived EVs were enriched from the total EV population based on their expression of membrane-bound placental alkaline phosphatase (PLAP). A quantitative, information-independent acquisition (sequential windowed acquisition of all theoretical mass spectra [SWATH]) approach identified and quantified the placental EV protein contents. PLAP^+^ EVs did not change in characteristics (size, shape, and markers) but did differ in numbers across gestation with low levels in PTB. A comparison analysis between the PLAP^+^ EV proteome from term and PTB revealed 96 proteins differing significantly (*P* < 0.05, false discovery rate 1%) across gestation. Bioinformatics analysis of differentially expressed proteins revealed consistent upregulation of inflammatory pathways in both upregulation of epithelial mesenchymal transition pathways at term and downregulation of coagulation/complement activation in preterm. Characterization of the proteomic profile in PLAP^+^ EVs across gestation demonstrates dramatic changes, which might be used to understand the biological process associated with early parturition and develop biomarkers for predicting high-risk status for PTB.

Preterm birth (PTB; birth before 37 weeks of completed gestation) is a major pregnancy complication that contributes to neonatal morbidity and mortality as well as chronic diseases later in adult life among the survivors (1,2). PTB in a mother also could cause serious maternal complications like hemorrhage, infection, intensive care unit admission apart from the long-term maternal cardiovascular morbidity and renal failure (3–5). The most common form of PTB is spontaneous PTB (sPTB) with unknown etiology. Approximately 60% of all PTBs are spontaneous, with little advancement in understanding the complete etiology of PTB (6–8). According to conventional theories of parturition, the initiation of signaling is primarily linked to fetomaternal endocrine and immune factors correlating with fetal growth and development. sPTB appears to be associated with the glucocorticoid signaling pathway, peroxisome proliferator–activated receptor γ, and interferon regulating factor 3 (9). The most significant changes identified from the total protein profile were related to complement activation with decreased level of complement C3 in the first trimester serum of women who delivered prematurely (10). Investigations also suggest that inflammatory and immune-related events in early pregnancy are part of the pathogenic mechanisms of sPTB (11). Placenta plays a major role of nourishing and providing protection to the developing fetus (12). Dysfunction in the placenta has been associated with adverse pregnancy outcomes (13). The unobtainability of this tissue during gestation restricts us from understanding its functions in the maintenance of the pregnancy through exchange of information between the mother and fetus. Propagation of various communication signals between fetomaternal compartments can occur either by endocrine or paracrine ways. Thus, maternal-fetal communication is essential for a healthy pregnancy, and recently our understanding of how cells can communicate with each other have undergone a shift with the identification of extracellular vesicle (EVs) and their role in maternal-fetal communication and intercellular signaling (14,15).

Recent advances in the field of EVs have implicated a role for small EVs called exosomes as potential mediators or carriers of signals between fetus and the mother (16–19). Exosomes are membrane-bound nanovesicles, with size ranging from around 50 to 150 nm and are shed by almost all cell types in the body (20–23). Exosomes carry bioactive molecules that include but are not limited to nuclear, cytosolic, and membrane proteins as well as lipids, messenger and micro ribonucleic acids (RNAs), and deoxyribonucleic acid, etc (19,21,24–27). The cargo carried as signaling molecules in the exosomes is packaged from the parent cell of origin following the process of exocytosis of the multivesicular bodies. Once the exosomes are released by exocytosis into the extracellular space, they travel from the place of origin to the distal organs along the systemic circulation (28). This has been reported in propagation of many types of diseases, especially cancers (29) as a form of nonhormonal communication or signaling. Interestingly, the exosomal cargo is a representative of the physiologic and or pathophysiologic state of the cell of origin, making it a good vector of paracrine signaling (30–34). Thus, the exosomal cargo represents a unique status of the organ or cell in circulation and acts as excellent footprints of the cellular signals.

Recently, we have identified that the micro RNA (miRNA) enclosed in circulating exosomes in the maternal blood represent a biomolecular “fingerprint” of the progression of pregnancy. Small RNA-Seq analysis reveals miRNA expression dynamics across PTB pregnancies. A total of 167 and 153 miRNAs were found to significantly change (*P* < 0.05) as a function of the gestational age across term and PTB pregnancies, respectively (24). Placental tissue-specific EVs have been studied as diagnostic markers for preeclampsia and other pregnancy outcomes (32,34,35). Several reports demonstrate that a major proportion of adverse pregnancy outcomes have their pathophysiologic origins at the uteroplacental interface, especially in early gestational periods (33,36,37). Additionally, we have recently reported the fetal-derived inflammatory signals generated by senescent fetal membrane (amniochorion) and placental cells at term (38). However, evidence gathered from all this literature has not effectively translated into our understanding of PTB pathways, suggesting that with all this knowledge, the signature of these signals and their precise mechanism in initiating parturition are still unclear.

Based on these reports and our success in isolating and characterizing fetal exosomes from maternal plasma samples, we hypothesized that placental-specific exosome cargo proteomic profiling will generate a descriptive roster of the secretome at various trimesters of pregnancy, both for term and preterm deliveries. This would depict the physiology of the placenta and provide an access to the development of the fetus/placenta in real time using minimally invasive blood samples. Therefore, profiling of differentially expressed proteins at different trimesters in term and preterm pregnancies will allow us to explore the usefulness of these proteins as biomarkers of high-risk pregnancies predictive of impending complications that could lead to early parturition. Using longitudinal samples from a well-characterized cohort of pregnant subjects from North India, our present analysis of placental-specific exosomes demonstrates the informative, longitudinal, placental, exosomal proteomic profile at various trimesters (first, second, and third) and during delivery in both term and preterm pregnancies.

## Methods

### Study group and biospecimen collection

A hospital-based cohort of pregnant women was initiated in 2015 at a district hospital in Gurugram, Haryana, (GCH), India, by the Pediatric Biology Center, Translational Health Science and Technology Institute of India as a unique collaborative interdisciplinary program among research institutes (National Institute of Biomedical Genomics, Kalyani; Regional Centre for Biotechnology, Delhi, NCR) and district (Gurugram Civil Hospital [GCH], Haryana) and tertiary care hospitals (Safdarjung Hospital, Maulana Azad Medical College, New Delhi). The study was approved by the Translational Health Science and Technology Institute of India Institutional Review Board (reference number THS 1.8.1, approval date 2/11/2015), and all methods were performed in accordance with the relevant guidelines and regulations. The objectives of this cohort were to identify the clinical, epidemiological, genomic, epigenomic, proteomic, and microbial correlates of PTB, discover molecular risk markers by using an integrative omics approach and generate a risk-prediction algorithm for PTB. Serial biospecimens were collected across pregnancy (at first, second, and third trimesters, respectively), at delivery, and after delivery. Ultrasound images were acquired serially during pregnancy, and the period of gestation was confirmed at enrollment by performing a dating ultrasound using standard fetal biometric parameters. The sample preparation at the study site was carried out in the research laboratory established at GCH on nationally accredited equipment, ISO /IEC 17025 and ISO 15189 accredited (National Accreditation Board for Testing and Calibration Laboratories). Plasma samples were stored at –80°C until analyses. All women provided written informed consent, and the study was approved by the Institute Ethics Review Board.

### Selection of samples for this study

The selection of participants for performing the proteomics of the placental-specific EVs was carried out using a nested case–control design (24). The cases and controls were selected from a defined population, composed of participants who had a normal singleton vaginal delivery, with no congenital abnormalities or associated comorbidity (preeclampsia, gestational diabetes, pregnancy-induced hypertension, medical condition complicating complication etc.) at any time during pregnancy. The cases were defined as participants who had delivered sPTB defined as <37 completed weeks period of gestation. Participants who delivered at >37 completed weeks to 40 completed weeks were considered controls. The cases and controls were matched to parity, gender of the baby, and month of delivery. We have previously established that EVs are stable when stored at –80°C (39), thus confirming the utility of stored plasma for EVs analysis.

### Enrichment of placental EVs from maternal blood

Exosomes were isolated from plasma as previously described (24,36,40). In brief, plasma was diluted with an equal volume of phosphate-buffered saline (PBS; pH 7.4) and centrifuged at 2000 x g for 30 minutes at 4^o^C (Sorvall, high-speed microcentrifuge, fixed rotor, Thermo Fisher Scientific Ins., Asheville, NC). The 2000 x g supernatant fluid was then centrifuged at 12000 x g for 45 minutes at 4^o^C (Sorvall, high-speed microcentrifuge, fixed rotor). The resultant supernatant fluid (2 mL) was transferred to an ultracentrifuge tube (Beckman, 10 mL) and centrifuged at 100000 x g for 2 hours (Sorvall, T-8100, fixed ultracentrifuge rotor). The 100000 x g pellet was suspended in PBS (10 mL) and filtered through a 0.22 μm filter (Steritop, Millipore, Billerica, MA) and then centrifuged at 100000 x g for 2 hours. Placental-derived EVs were enriched from the total EV population based on their expression of membrane-bound placental alkaline phosphatase (PLAP) as previously described (40). Briefly, anti-PLAP antibody (Abcam, ab118856) (41) was conjugated to protein-A agarose beads (Cell signaling, #9863) using the cross-linker disuccinimidyl suberate (Thermo Scientific # 21655). The antibody-conjugated beads were washed using 1x PBS and incubated overnight with the maternal plasma-derived exosomes at 4°C with rotation. After incubation, the samples were briefly centrifuged, and the supernatant from each tube was reserved (PLAP^–^ exosomes), followed by the washing of the beads with 100 μL of 1x PBS. Bound PLAP^+^ exosomes were eluted from the beads by the addition of 0.1 M glycine-hydrochloric acid, pH 2.8. After a brief centrifuge, the supernatant containing the eluted exosomes was transferred to tubes containing 1 M Tris, pH 8.0, to neutralize the pH. The PLAP enrichment process has been validated previously using recombinant PLAP as a control (42,43).

### Nanoparticle tracking analysis

Nanoparticle tracking analysis (NTA) was performed using a NanoSight NS500 instrument (NanoSight NTA 2.3 Nanoparticle Tracking and Analysis Release Version Build 0033) following the manufacturer’s instructions (44). Samples were diluted 1 in 1000 with Dulbecco’s PBS to obtain between 10 to 100 particles per image (optimal ∼50 particles per image). The NS500 instrument measured the rate of Brownian motion of nanoparticles in a light-scattering system that provides a reproducible platform for specific and general nanoparticle characterization (NanoSight Ltd., Amesbury, United Kingdom). Suspended nanoparticles were exposed through the laser beam that traversed the sample chamber and scattered the light, discerning the nanoparticles through a magnification microscope. The samples were mixed before introduction into the chamber (temperature: 25°C) and the camera level set to obtain an image that has sufficient contrast to clearly identify particles while minimizing background noise to a video recording (camera level: 10, capture duration: 30 seconds). The captured videos (2 videos per sample) were then processed and analyzed. Each video file was processed and analyzed to give the mean and mode of particle sizes, along with the concentration on the number of particles. An Excel spreadsheet was automatically generated and data was imported into Graph Pad-Prism7 (GraphPad Software, San Diego, CA).

### Fluorescence NTA

Qdots (Qdot nanocrystals) were conjugated to anti-CD63 (45), which is mainly associated with membranes of intracellular vesicles and, thus, is widely used as a marker of small EVs such as exosomes, PLAP, or immunoglobulin (Ig) G1 isotype control antibody (IgG1 sc-34665, Santa Cruz Biotechnology) (45) with a SiteClick Qdot 605 Antibody Conjugation Kit (Life Technologies), executed according to the manufacturer’s instructions as previously described (42,46). Exosomes were diluted in PBS and incubated with FcR blocking reagent (10 µL, 10 minutes at 4°C) (MACS Miltenyi Biotec), followed by incubation with anti-CD63-Qdot605, PLAP–Qdot605, or IgG1-Qdot605 (10 µL, 1:100) for 30 minutes in the dark at room temperature. Samples (ie, [1] exosomes alone, [2] exosomes + IgG1-Qdot605, [3] exosomes + anti-CD63-Qdot605 and exosomes + anti-PLAP-Qdot605, [4] background controls, [5] FcR blocking reagent + IgG1-Qdot605, [6] FcR blocking reagent + anti-CD63-Qdot605, and [7] FcR blocking reagent + anti-PLAP-Qdot605) were then diluted to 500 µL with PBS and analyzed using the NanoSight NS500 instrument and NTA software (Malvern Panalytical Ltd., Malvern, UK). In fluorescence mode (ie, camera level 9, shutter speed 11.25 ms and slider gain 250), five 60-second videos were captured for each sample and analyzed.

### Sequential window acquisition of all theoretical mass spectrometry

#### Ion library generation. 

To generate the ion library used in the sequential windowed acquisition of all theoretical mass spectra (SWATH) mass spectra analysis, exosome samples derived from maternal plasma were reduced, alkylated, and trypsinized using an in-gel digestion method. In brief, the EV samples were first mixed with Bolt™ LDS sample buffer (ThermoFisher), sonicated for 5 minutes and heated at 72°C for 10 minutes. Samples were then separated based on the molecular weight on a Bolt Bis-Tris Plus polyacrylamide gel (ThermoFisher) at 200 V until full separation. The resulting gel was stained with Simply Blue for 1 hour. Ten gel bands were excised from each sample lane and were destained in 50% acetonitrile (v/v) to 50 mM ammonium bicarbonate (ABC). The cysteine bonds were reduced with 10 mM dithiothreitol (Sigma) in 100 mM ABC at 56°C for 30 minutes and alkylated with 50 mM iodoacetamide (Sigma) for 20 minutes in the dark. Trypsin solution at 20 ug/mL (Promega) in 50 mM ABC were added to just cover the gel pieces before an overnight incubation at 37°C. The resulting tryptic peptides were desalted using a C18 96-well plate (Biotage ISOLUTE), according to manufacturer’s instruction.

### Filter-aided sample preparation

PLAP-enriched exosomes were digested using the filter-aided sample preparation as previously described (47). Samples were lysed using 4% sodium dodecyl sulfate in 100 mM Tris, pH 8.5. Samples were sonicated and heated at 95^o^C for 5 minutes and then mixed with 200 μL of 8 M urea/50 mM Tris, pH 8.0. This mixture was transferred onto a 30 kDa molecular weight cutoff filter device (Pall Nanosep), which was used to concentrate the protein lysate using a series of washings by centrifugations. Proteins were reduced with 100 mM DTT for 1 hour in room temperature and alkylated with 50 mM iodoacetamide for 20 minutes in the dark. Reagents were removed by centrifugation at 10000 x g at 20°C for 5 minutes. The concentrate was then subjected to trypsin digestion (enzyme to protein ratio 1:50, 50 mM ABC) at 37°C for overnight. The digests were collected in a fresh tube by centrifugation, and the filter device was rinsed with 50 μL of 0.5 M sodium chloride and centrifuged again. The resulting tryptic peptides were desalted using a C18 96-well plate (Biotage ISOLUTE) according to manufacturer’s instruction. The eluted peptides were dried and resuspended in 40 μL 0.1% formic acid to be subjected to mass spectrometry.

### Mass spectrometry data acquisition

Tryptic peptides were separated using an Eksigent NanoLC system coupled with a ReproSil-Pur Basic-C18-HD, 5-µm column over a 90-minute gradient ranging from 2% to 35% (buffer A: 0.1% formic acid [v/v]; buffer B: 100% acetonitrile, 0.1% [v/v]) formic acid. The resulting peptide samples were processed using information-dependent acquisition on an AB Sciex 5600 TripleTOF mass spectrometer with the top 20 precursor ions automatically selected for fragmentation (15).

For SWATH acquisition, the 5600 Triple TOF was operated in a looped product ion mode. Using an isolation width of 26 m/z, a set of 32 overlapping windows (1 m/z overlap) was constructed covering the mass range 400 to 1200 m/z.

### Data processing

ProteinPilot version 4.5b software and the Paragon Algorithm were used to search against a human SwissProt database. A global false discovery rate (FDR) of 1% was used as the threshold for the number of proteins for import. For SWATH processing, the SWATH Acquisition Microapp (version 2.0) within PeakView (version 2.2) was used (48). Within the Microapp, a setting of 3 peptides per protein, 4 transitions per peptide, peptide confidence threshold corresponding to 1% global FDR, and FDR threshold of 1% was used. The retention time was then manually realigned with a minimum of 5 peptides with constantly high signal intensities and distributed along the time axis. The resulting peak area for each protein after SWATH processing was exported to MakerView (version 1.3.1) and the resulting data was normalized using the most likely ratio method.

### Linear mixed model analysis

Normalized protein expression was subjected to linear mixed modeling using the lme4 package (version 1.1.15) in R (49). Statistical analysis (likelihood ratio test) was performed comparing the full model, which included the gestational age variable, compared with a simpler model without the gestational age variable. *P* <0.05 was the cutoff for statistical significance. Afterwards, the normalized expression for statistically significant proteins was scaled between 0 and 1, before hierarchal clustering using Euclidean distance analysis to determine proteins with similar trends in expression across gestation. These data were plotted as a circular cladogram, which was generated using the ggtree package (version 1.10.2), while graphs were generated using ggplot2 package (version 2.2.1) in R (50).

### Ingenuity Pathway Analysis of identified proteins

Pathway enrichment analyses were performed with Ingenuity Pathway Analysis (IPA, Qiagen, Hilden, Germany) (51). IPA was performed to identify canonical pathways, diseases and functions, and protein networks. Heat map analysis was used to demonstrate the expression patterns of canonical pathways based on Z-scores. Significantly enriched pathways for the proteins and pathways were identified with the criterion of *P* <0.05.

### Gene set enrichment analysis

To determine the genes associated with changes in the protein profile within circulating placental vesicles, Gene Set Enrichment Analysis (GSEA, version 3.0) was performed (52). Normalized SWATH results from exosomes isolated from plasma obtained from term and PTB across pregnancy were used in the GESA. The protein expression data were processed using the hallmark gene sets within the MSigDB database v6.2 with permutations set at 1000 and Signal2Noise metric for ranking genes. Default values were chosen for all other parameters.

### Statistical analysis

Data are presented as mean ± SEM, with n = 20 (term controls) and n=10 (sPTB) different patients per group (ie, first, second, and third trimester; total samples for sPTB group = 30 and for the term group = 60). The effects of gestational age on number of vesicles (ie, vesicles <50 nm, 50–150 nm, 150–200 nm, >200 nm, total CD63^+ve^ and PLAP^+ve^) and proteomic profile were assessed using repeated measures of 2-way analysis of variance, with the variance partitioned between gestational age and condition; thus, gestational age was treated as an independent factor. Statistical differences between groups were identified by post hoc analyses of Bonferroni tests to compare sPTB with term pregnancy. For 2-group analyses (paired data), Student *t* tests were used to assess statistical difference. Statistical significance was defined as at least *P* < 0.05. Statistical analyses were performed using commercially available packages (Stata 11, StatCorp, College Station, Texas USA and Prism 6, GraphPad Inc, La Jolla, CA 92037). Proteomic analysis results are summarized in Table 1. All identified proteins and supplemental materials were submitted to massive.ucsd.edu (53).

**Table 1.** Protein changes within circulating exosomes PLAP^+ve^ in A) across term pregnancy, B) across PTB pregnancies, and C) comparison between term and PTB across pregnancy, identified by information-dependent acquisition (IDA) and sequential windowed acquisition of all theoretical mass spectra (SWATH) were submitted to the Ingenuity Pathway Analysis (IPA) to determine the main signaling pathways associated with the protein profile within circulating placental exosomes.

*A)Changes in exosomes PLAP+ve across term pregnancies*		*B)Changes in exosomes PLAP+ve across PTB pregnancies*		*C)Changes in exosomes PLAP+ve between term and PTB across pregnancy*	
Top Canonical Pathways	*pvalue range / #molecules*	Top Canonical Pathways	*pvalue range / #molecules*	Top Canonical Pathways	*pvalue range / #molecules*
* Acute Phase Response Signaling*	*6.10E-08*	*LXR/RXR Activation*	*6.55E-15*	*LXR/RXR Activation*	*6.43E-06*
* LXR/RXR Activation*	*3.10E-05*	*FXR/RXR Activation*	*4.89E-13*	*FXR/RXR Activation*	*7.83E-06*
**Top Upstream Regulators**		**Top Upstream Regulators**		**Top Upstream Regulators**	
* IL-1*	*2.73E-09*	*dexamethasone*	*2.36E-06*	*rosiglitazone*	*2.00E-07*
* L-triiodothyronine*	*1.15E-06*	*nitrofurantoin*	*2.45E-06*	*dexamethasone*	*3.07E-07*
**Top Diseases and Disorders**		**Top Diseases and Disorders**		**Top Diseases and Disorders**	
* Inflammatory Response*	*5.96E-03 - 1.62E-12 / 42*	*Inflammatory Response*	*8.68E-03 - 6.55E-09 / 32*	*Inflammatory Response*	*3.86E-03 - 1.28E-08 /31*
* Metabolic Disease*	*5.96E-03 - 1.62E-12 /24*	*Cardiovascular Disease*	*9.01E-03 - 1.70E-08 / 13*	*Dermatological Diseases and Conditions*	*2.17E-03 - 7.12E-07 /17*
**Top Molecular and Cellular Functions**		**Top Molecular and Cellular Functions**		**Top Molecular and Cellular Functions**	
* Cellular Function and Maintenance*	*5.96E-03 - 3.61E-14 / 23*	*Cellular Function and Maintenance*	*7.73E-03 - 7.26E-10 / 20*	*Cellular Movement*	*4.10E-03 - 4.94E-10 /23*
* Cellular Movement*	*5.96E-03 - 1.41E-12 / 29*	*Cellular Compromise*	*7.32E-03 - 6.55E-09 /22*	* Cellular Function and Maintenance*	*4.12E-03 - 4.68E-09 /18*
**Top Physiological System Development and Function**		**Top Physiological System Development and Function**		**Top Physiological System Development and Function**	
* Immune Cell Trafficking*	*5.96E-03 - 1.41E-12 / 25*	*Humoral Immune Response*	*4.88E-03 - 3.67E-07 / 8*	*Immune Cell Trafficking*	*4.34E-03 - 4.94E-10 / 19*
* Humoral Immune Response*	*3.42E-09 - 1.62E-12 / 10*	*Immune Cell Trafficking*	*8.68E-03 - 1.25E-06 / 18*	* Hematological System Development and Function*	*4.34E-03 - 3.82E-07 /20*

## Results

### Description of the participants

A hospital-based cohort of pregnant women was studied at a district hospital in Gurugram, Haryana, (GCH), India. Serial biospecimens were collected across pregnancy (at first, second, and third trimesters, respectively), at delivery and after delivery. The detailed characteristics of this cohort are described in our prior publication (24). Supplemental Table 1 describes the comparison between the demographic and the clinical characteristics of the cases and controls. No significant differences between various sociodemographic parameters were seen between cases and controls.

### Quantification of total and placenta-derived EVs present in maternal circulation

**Figure 1.**
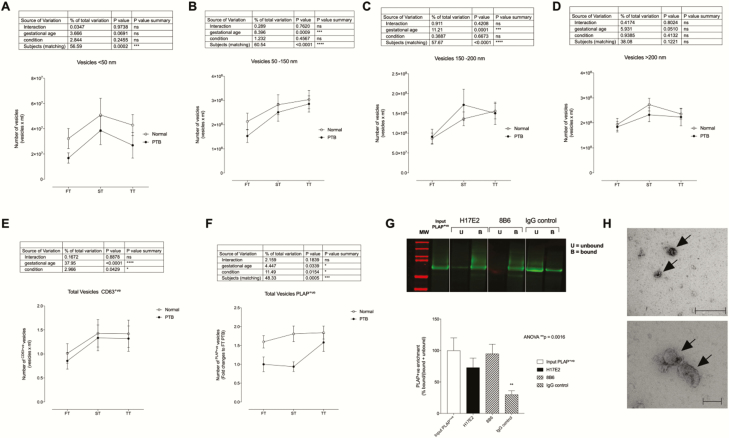
Analysis of extracellular vesicles across gestation. Enrichment of small vesicles was performed from plasma samples obtained from term and preterm birth (PTB) pregnancies throughout gestation. The presence of extracellular vesicles (EVs) of different sizes was evaluated by nanoparticle tracking analysis in light scatter and fluorescence mode to determine the presence of placental EVs in maternal circulation. (A) Vesicles <50nm, (B) vesicles between 50 and 150 nm, (C) vesicles between 150 and 200 nm, (D) vesicles >200 nm, (E) vesicles CD63^+ve^ profile across gestation, (F) vesicles placental alkaline phosphatase (PLAP)^+ve^ across gestation, (G) representative western blot gel with PLAP antibody; H17E2 and 8B6-PLAP clones, (H) electron micrograph indicating the morphology and size of exosomes. ns, not significant.

The gestational age variation of different populations of EVs based on their size (ie, <50 nm, 50–150 nm, 150–200 nm, and >200 nm) were analyzed by 2-way repeated measures of analysis of variance with the variance partitioned between gestational age, condition, and subject (Fig. 1). A significant effect by subject (ie, patient variation indicating that the levels of EVs were different across the women) was identified in all EV subpopulations (Fig. 1A-C) (*P* < 0.0002) except for vesicles >200 nm (Fig. 1D). A significant effect as a function of the gestational age was also identified in EV subpopulations between 50 and 150 nm and 150 and 200 nm (Fig. 1B and C) (*P* < 0.0009). No difference in different population of EVs between term and PTB (ie, condition) were observed (*P* > 0.05). To note, placental EV markers and morphology were not different between gestational ages in both term and preterm gestations.

We analyzed the total number of EVs (Qdot-CD63^+^) and placenta-derived EVs (Qdot-PLAP^+^) present in the maternal circulation using fluorescence nanoparticle tracking analysis (Fig. 1E and F). A significant effect by subject (ie, patient variation) and gestational age were identified (*P* < 0.0002) in the total circulating CD63^+^ EVs (Fig. 1E), without any significant difference between term and PTB across gestation (ie, condition). Interestingly, the concentrations of circulating placental EVs (PLAP^+^) were significantly different between term and PTB across gestation (Fig. 1F), with a significant effect by subject (ie, patient variation; *P* < 0.001) and gestational age (*P* < 0.05). This result suggests that changes in the concentration of PLAP^+^ EVs in maternal circulation might be useful as a biomarker to predict PTB. Therefore, we isolated the PLAP^+^ EVs to profile their protein content by mass spectrometry. We used 2 commercial antibodies for the enrichment of placental EVs from the total circulating EVs present in maternal circulation (Fig. 1G). Using the PLAP clones H17E2 and 8B6, a 73% [15] and 95% [17] enrichment [SEM] was obtained, respectively, and was significantly higher compared with the IgG control (30 ± 6% (Fig. 1G). Finally, transmission electron microscopy of enriched placental EVs from total circulating EVs demonstrated the cup‐shaped morphology and lipid bilayer typically associated with EVs (Fig. 1H).

### Placental EVs protein signature across normal pregnancy

**Figure 2.**
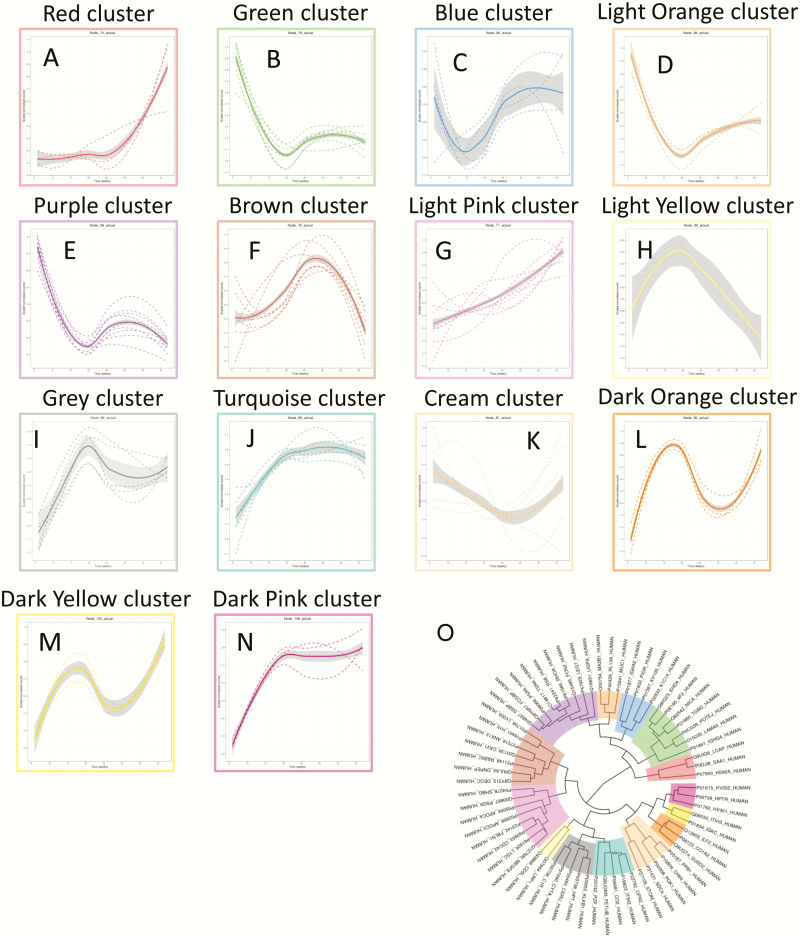
Changes in the protein profile in placental exosomes present in maternal circulation across normal gestation. Linear mixed modeling of all statistically significant proteins that change across gestation was performed for normal pregnancies. Linear mixed modeling in normalized protein abundance was performed on all of the statistically significant proteins (*P* < 0.05) that change across gestation for normal pregnancies, using the lme4 package in R. The data were scaled between 0 and 1 before hierarchical clustering analysis using Euclidean distance, which is displayed as a circular cladogram (generated using the ggtree package in R). Each color of the circular cladogram (O) represents a different cluster and its trend, as shown in (A) through (N). The y-axis shows scaled normalized counts, and the x-axis shows gestation age in weeks (5– 40 weeks).

**Figure 3.**
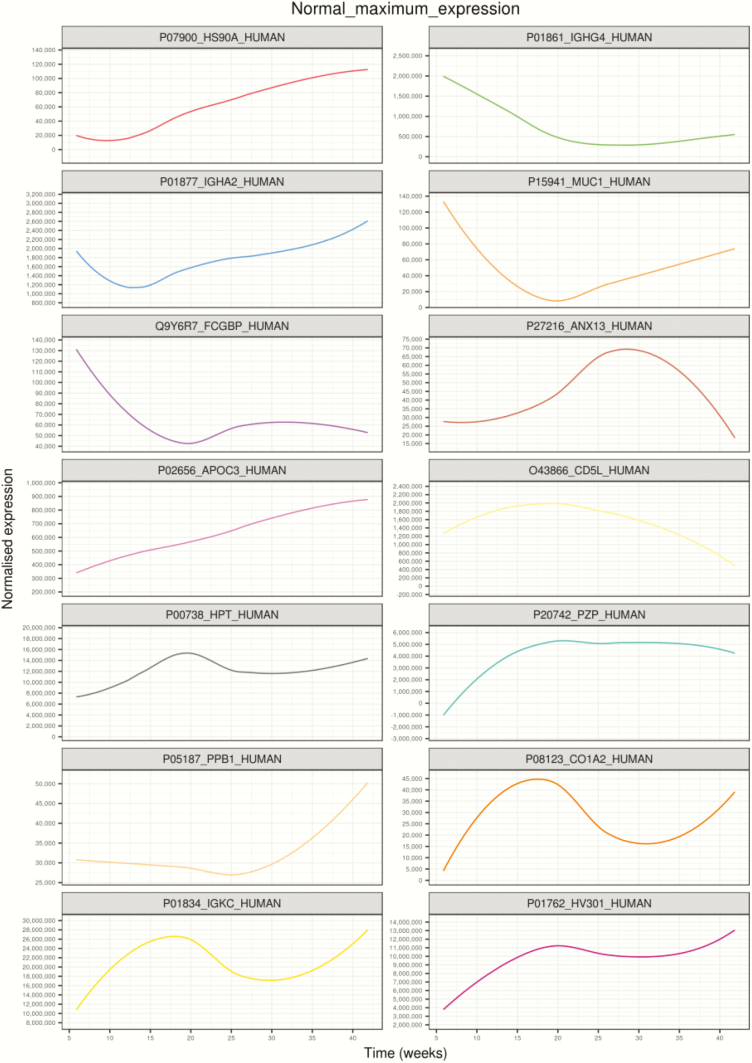
Highest expressed proteins in each cluster identified using linear mixed modeling for normal pregnancies across gestation. Linear mixed modeling and circular cladogram analysis for normal pregnancies across gestation were divided into 14 clusters. The highest expressed protein from each cluster was extracted and color coded based on the cluster of origin. Clusters are shown in ascending order, corresponding to (A) through (N) in Fig. 2, starting from the top of the figure, reading from left to right, and continuing onto the next line of graphs below.

A total of 66 proteins across gestation in term pregnancies revealed significant changes (*P* < 0.05) Supplemental Table 2. Hierarchical clustering analysis of the average protein expression profiles across gestation revealed a variety of trends (Fig. 2). Specifically, trends that increased to a maximum peak expression in the third trimester were clusters A, G, J, M, and N; trends that rapidly decreased in expression until the end of the first trimester then increased to a peak at the end of the second trimester were clusters B, D, and E. An increasing trend throughout gestation was identified in cluster G; a trend that rapidly decreased in expression until the end of the first trimester then increased in expression until the end of the third trimester was observed for cluster K. A trend that increased rapidly from the beginning of the third trimester was cluster A. A trend that increased until the end of the first trimester then declined steeply until delivery was observed for cluster H. An increase until the end of the first trimester then a gradual decline was observed in cluster I. An increase was observed at the end of the first trimester and later a decrease in the early third trimester was observed, followed by an increase until delivery in cluster L. Proteins with the largest maximum expression in each cluster are presented in Fig. 3.

### Placental EVs protein signature across PTB pregnancy

**Figure 4.**
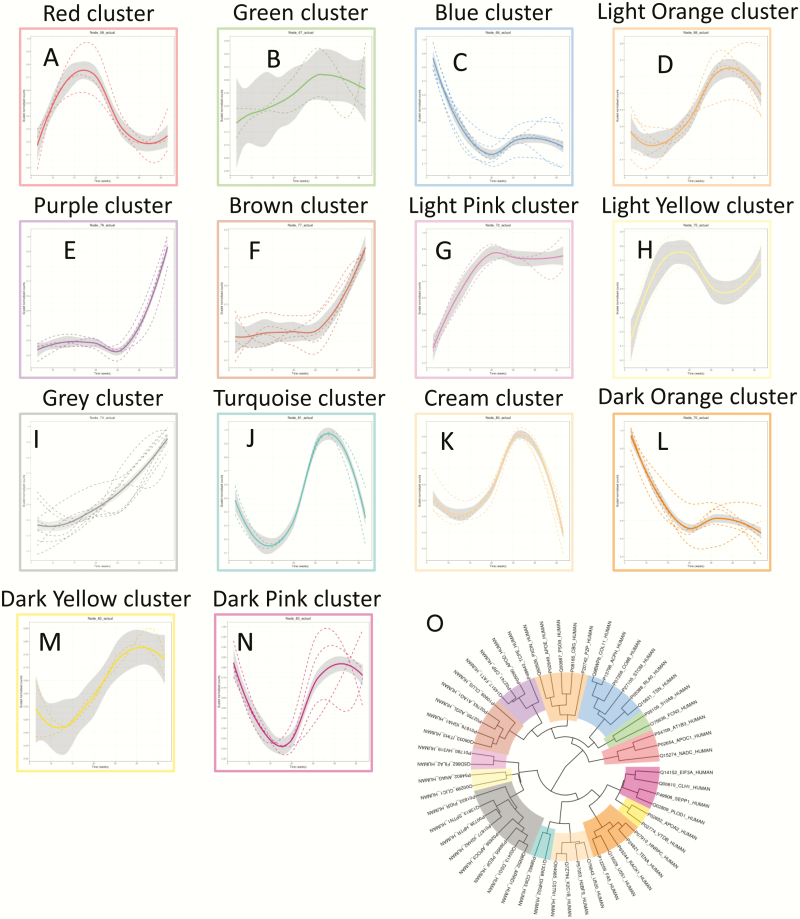
Changes in the protein profile in placental exosomes present in maternal circulation across preterm birth gestation. Linear mixed modeling of all statistically significant proteins that change across gestation for preterm birth (PTB) pregnancies was performed. Linear mixed modeling in normalized protein abundance was performed on all of the statistically significant proteins (*P* < 0.05) that change across gestation for normal pregnancies, using the lme4 package in R. The data were scaled between 0 and 1 before hierarchical clustering analysis using Euclidean distance, which is displayed as a circular cladogram (generated using the ggtree package in R). Each color of the circular cladogram represents a different cluster and its trend, as shown in (A) through (N).

**Figure 5.**
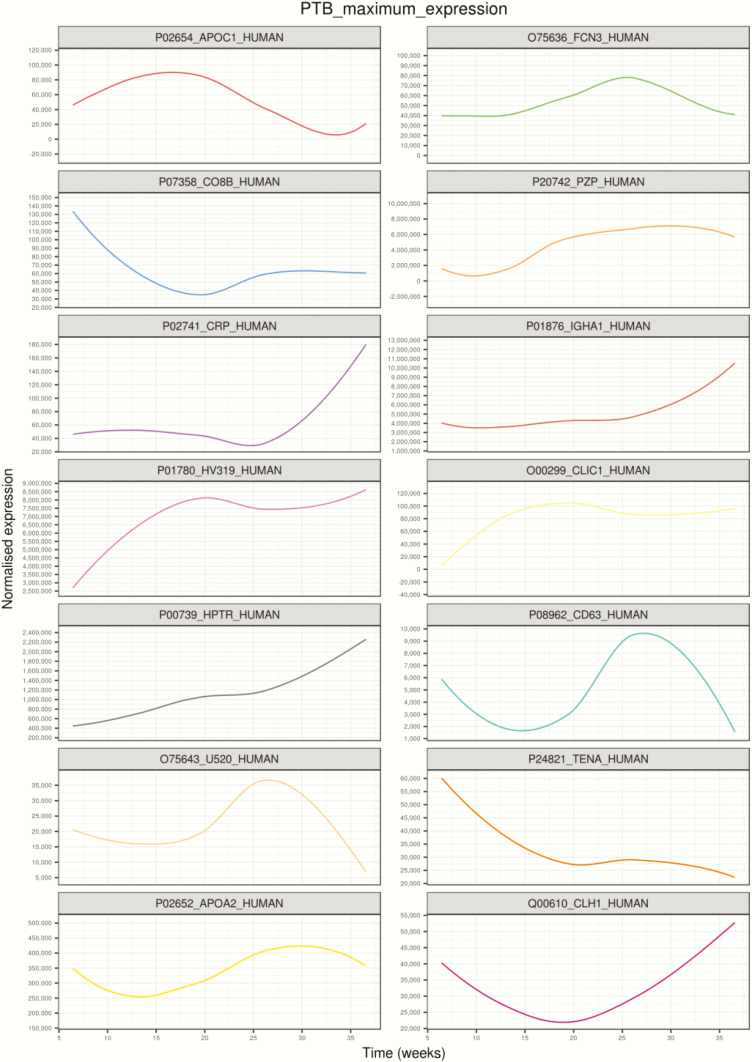
Highest expressed proteins in each cluster identified using linear mixed modeling for preterm birth pregnancies across gestation. Linear mixed modeling and circular cladogram analysis for preterm (PTB) pregnancies across gestation were divided into 14 clusters. The highest expressed protein from each cluster was extracted and color coded based on the cluster of origin. Clusters are in ascending order, corresponding to (A) through (N) in Fig. 2, starting from the top of the figure, reading from left to right, and continuing onto the next line of graphs below.

A total of 54 proteins across gestation in PTB pregnancies demonstrated significant changes (*P* < 0.05) Supplemental Table 3. Hierarchical clustering analysis of the average protein expression profiles across gestation revealed a variety of trends (Fig. 4). Specifically, trends that had peak expression in the second trimester were clusters A and H; a trend that had peak expression at the beginning of the third trimester was identified in cluster D, J, K, and M. Decreasing trends in expression across gestation were observed for clusters C. Gradual increase until the end of third trimester and then a slight decrease at delivery was observed in cluster B. Cluster G showed an increase early in the second trimester and then remained constant until delivery. Clusters E and F revealed peaks in expression at delivery. Gradual increase throughout gestation and peak at delivery was observed in cluster I. A rapid decline until the end of first trimester and then a peak at the end of the third trimester with a slight decrease during delivery was observed in cluster N. Proteins with the largest maximum expression in each cluster are presented in Fig. 5.

### Differential protein abundance of placental EVs across gestation in normal compared with PTB pregnancy

**Figure 6.**
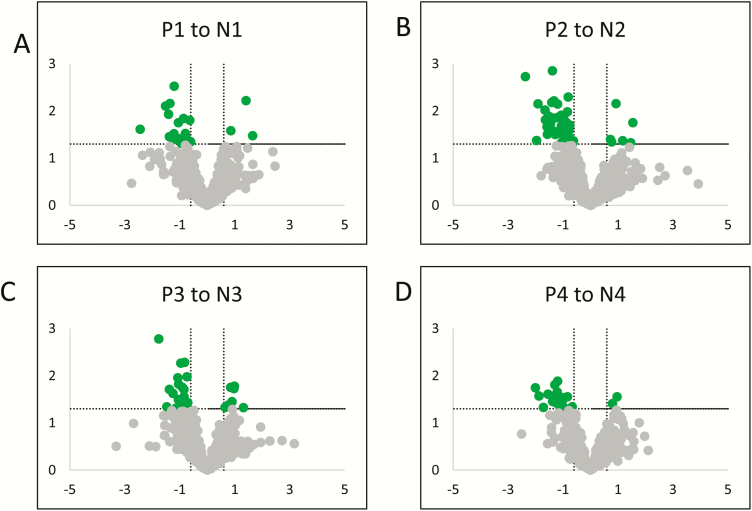
Volcano plots for P1 vs N1, P2 vs N2, P3 vs N3, and P4 vs N4. Statistically significant proteins (adjusted *P* < 0.05) with a log2 fold change ±0.6 are represented by green dots. Differentially expressed proteins that did not reach statistical significance are represented by gray dots. P is preterm; N is normal; 1 to 3 is first through third trimester and 4 is laboring.

A total of 96 proteins were found to significantly change (*P* < 0.05) across gestation for normal pregnancies compared with PTB pregnancies (Supplemental Table 4). A comparison analysis on the variation in the relative abundance of proteins within placental EVs between selected pair groups (ie, PTB first trimester vs term first trimester, PTB second trimester vs term second trimester, PTB third trimester vs term third trimester, and preterm labor vs term labor) was established and presented as a volcano plot (Fig. 6A-6D). Proteomic analysis from all individual exosome samples identified 720 differentially expressed proteins across all groups (Supplemental Table 5). A total of 23, 51, 29, and 24 statistically significant protein identifications were made (*P* < 0.05) in the relative expression of exosomal proteins in PTB first trimester vs term first trimester, PTB second trimester vs term second trimester, PTB third trimester vs term third trimester, and preterm labor vs term labor, respectively.

**Figure 7.**
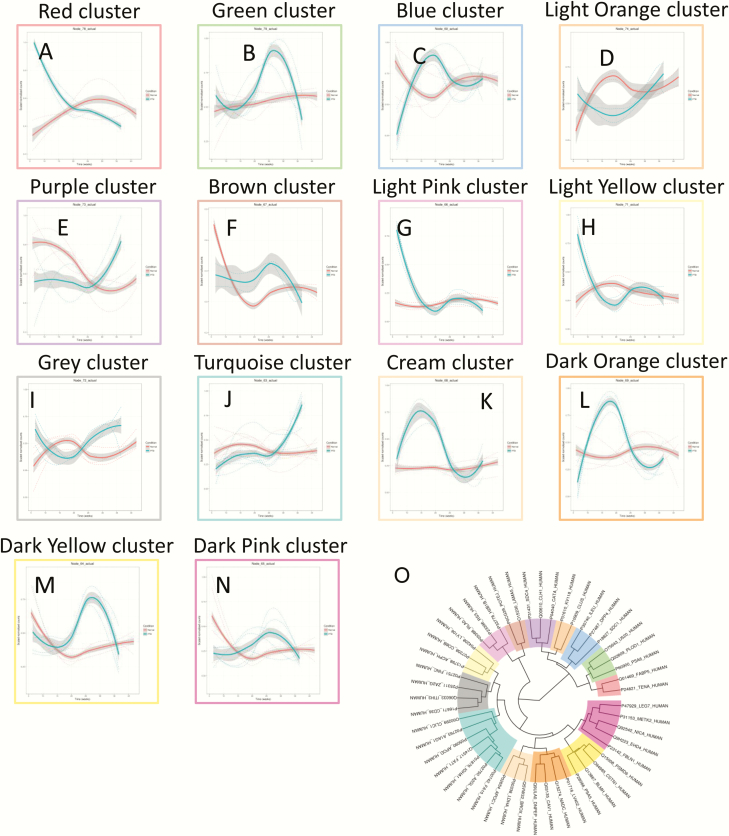
Changes in the protein profile in placental exosomes present in maternal circulation across gestation in preterm birth compared with normal pregnancies. Linear mixed modeling of all statistically significant proteins that change across normal pregnancy compared with preterm birth (PTB). Linear mixed modeling in normalized protein abundance was performed on the all statistically significant proteins (*P* < 0.05) that change across gestation for normal pregnancy compared with PTB, using the lme4 package in R. The data were scaled between 0 and 1 before hierarchical clustering analysis using Euclidean distance, which is displayed as a circular cladogram (generated using the ggtree package in R). Each color of the circular cladogram represents a different cluster and its trend, as shown in (A) through (N).

**Figure 8.**
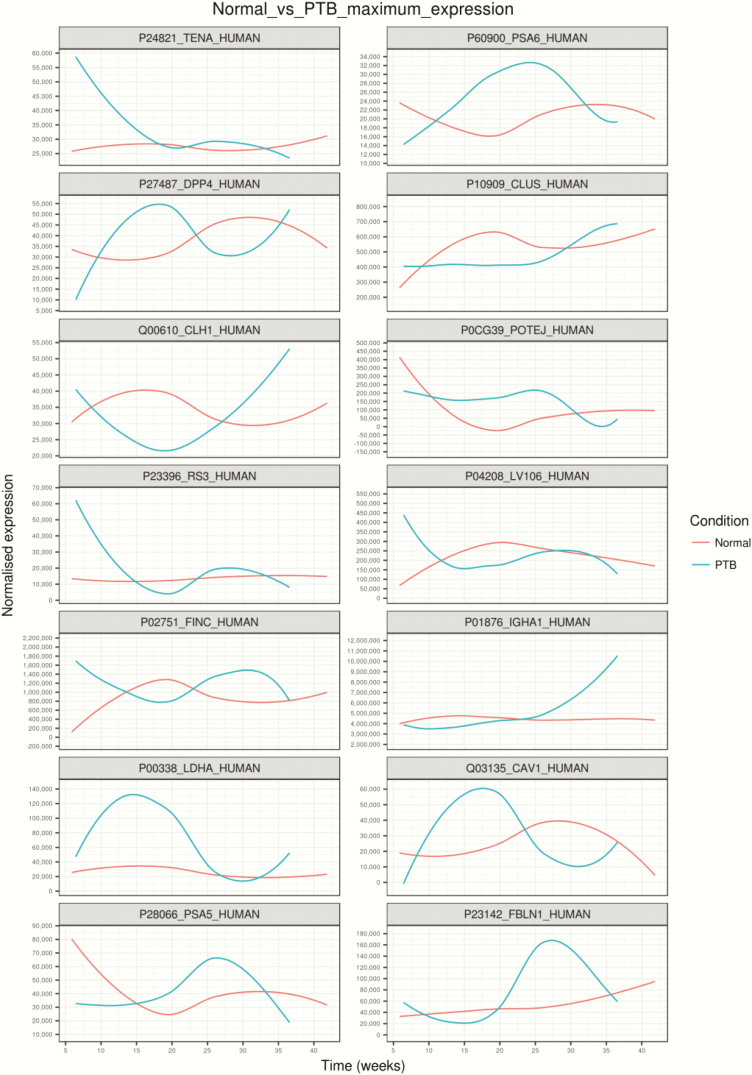
Gene Set Enrichment Analysis of the protein profile in circulating placenta-derived exosomes. Highest expressed proteins in each cluster are identified using linear mixed modeling for normal compared with preterm birth (PTB) pregnancies across gestation. Linear mixed modeling and circular cladogram analysis for protein in placental extracellular vesicles (EVs) that changes in normal pregnancy compared with PTB across gestation were divided into 14 clusters. The highest expressed protein from each cluster was extracted and color coded based on the cluster of origin. Clusters are in ascending order, corresponding to (A) through (N) in Fig. 2], starting from the top of the figure, reading from left to right, and continuing onto the next line of graphs below.

Hierarchical clustering analysis of the average protein expression profiles across gestation revealed a variety of trends (Fig. 7). Specifically, trends that had the largest difference in expression within the first trimester comparing normal with PTB pregnancies were clusters A, C, G, and H; trends that had the largest difference in expression within the second trimester comparing normal with PTB pregnancies were clusters B, C, D, K, L, F, M, and N; trends that had the largest difference in expression within the third trimester comparing normal with PTB pregnancies were clusters E, I, and J. Proteins with the largest maximum expression in each cluster are presented in Fig. 8.

### IPA

**Figure 9.**
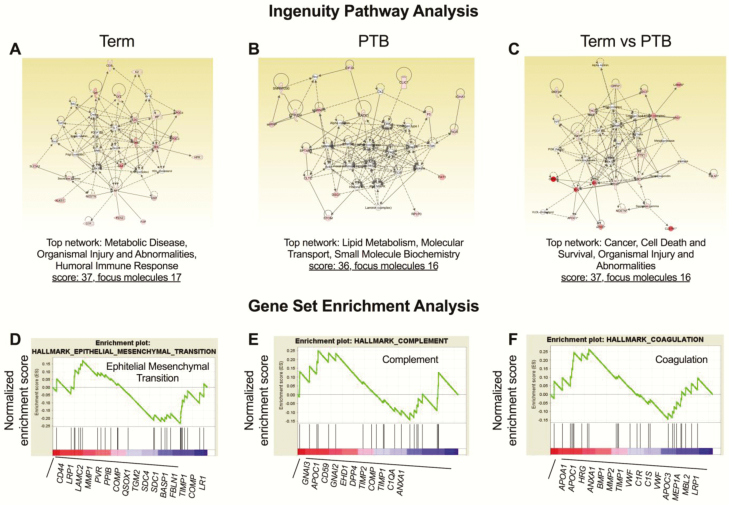
Bioinformatic analysis of the protein profile in circulating placenta-derived exosomes. Ingenuity pathways analysis (IPA) of the exosomal proteomic profile in term and preterm birth (PTB) was used to create a network analysis showing molecules with functions in term pregnancies related to (A) Metabolic Disease, Organismal Injury and Abnormalities and Humoral Immune Response; in PTB pregnancies (B) Lipid Metabolism, Molecular Transport, and Small Molecule Biochemistry; and term pregnancies compared with PTB (C) Cancer, Cell Death and Survival, Organismal Injury and Abnormalities. Identification of the top candidate protein sets enriched in circulating placental exosomes from term and PTB across gestation were identified by gene set enrichment analysis (GSEA). GSEA ranked the proteins in each group based on their differential expression level. Each subset of protein was evaluated using the normalized enrichment score (the green line). Black vertical lines mark the positions where the members of a particular pathway appear in the ranked list of genes. The proteins that contributed most to the enrichment score are listed below the plot. The most upregulated genes are on the left (red), and the downregulated genes are on the far right (blue). Enrichment score (ES) and normalized enrichment score (NES) for (D) Epithelial Mesenchymal Transition (ES = –0.23; NES = –0.83), (E) Complement (ES = 0.29; NES = 0.95), (F) Coagulation (ES = –0.26; NES = –0.84).

Using IPA to analyze the network of protein interactions, we demonstrated that the protein profile in EVs from term and PTB regulated signaling pathways associated with metabolic disease and lipid metabolism, respectively (Fig. 9A and 9B). Interestingly, analysis of the differences in the protein profile in the placenta-derived exosomes between term and PTB across gestation suggests a regulation in cell death and survival represented as top network (Fig. 9C). Next, to determine whether the differences in the protein profile in the circulating placenta-derived EVs were associated with changes in gene expression, a GSEA was used. GSEA of the total cell protein revealed several gene sets that were significantly enriched in circulating placental exosomes across gestation in term and PTB pregnancies (Fig. 8D, 8E and 8F). This was demonstrated by the normalized enrichment score, which is the primary statistic examining whether a gene set is overrepresented in a ranked list of genes, with FDR < 0.01. Of particular interest was an enrichment of proteins involved in epithelial mesenchymal transition, complement, and coagulation.

To investigate the potential functions of the differentially expressed proteins, IPA of the PLAP^+^ EVs proteomic profile per trimester pair (ie, first trimester preterm vs first trimester normal term, second trimester preterm vs second trimester normal term, third trimester preterm vs third trimester normal term, preterm labor vs normal labor) was performed (see Supplemental Figure 1 for IPA output). As shown in Fig. 9A, the heat map of Z-scores indicated differences observed in the top 20 biological functions between group analyses. Coagulation and complement activation were downregulated in preterm compared with normal pregnancies, whereas other nonspecific inflammatory pathways were upregulated in preterm compared with normal pregnancies. In Fig. 9B, the top 20 biological functions of PLAP^+^ exosomes were identified. Inflammatory pathways were consistently upregulated in preterm compared with normal term deliveries during early and late pregnancy, while coagulation was downregulated in all preterm compared with normal pregnancies. This data demonstrates that during pregnancy, circulating exosomes derived from the human placenta are packaged with a specific set of proteins, which differs in term compared with PTB.

## Discussion

In this study, placental EVs circulating in maternal blood were isolated and enriched by immunoaffinity capture using anti-PLAP–coated beads. Protein profile changes in circulating placental EVs isolated across gestation in term and sPTB pregnancies with intact membranes were characterized in a time series manner. An innovative approach was implemented in this study using linear mixed modeling to determine protein expression as a function of gestational age in term and sPTB pregnancies. The data obtained identified specific PLAP^+^–EV protein changes in term and sPTB as a function of the gestational age during pregnancy. Bioinformatics analysis revealed unique canonical pathways and biological functions represented in circulating placental EVs in maternal blood. Interestingly, the protein profiles of placental EVs in term pregnancies were associated with nonspecific inflammation and epithelial mesenchymal transition signaling pathways. However, sPTB was characterized by nonspecific inflammation but not with epithelial mesenchymal transition signaling pathways. Additionally, coagulation and complement activation-related proteins were downregulated in sPTB.

While there is substantial interest in identifying the role of placenta-derived EVs under normal and pathological pregnancies and their utility as biomarkers and therapeutic interventions, progress in the field is hindered by the inability to isolate placental EVs from the maternal circulation. Several studies have reported changes in the quantities and activity of placental EVs during normal and abnormal pregnancies using a wide range of experimental procedures. Placental perfusion has been used to isolate EVs in the context of preeclampsia (PE) (54–59) in which higher levels of placental EVs in PE compared with healthy pregnancies have been identified. Similarly, the levels of placental EVs are more elevated in plasma obtained from PE across gestation (ie, longitudinal study design) compared with healthy pregnancies (47,60), and the levels of small EVs are higher in GDM compared with normal pregnancies (61). Subpopulations of EVs have been isolated from placental explants and established changes in their proteomic cargo across the nano, micro, and macrovesicles preparations (5). Other studies have utilized cell-conditioned media from primary placental cells (62,63) or placental cell lines (13) and identified changes in EV release and bioactivity on target or recipient cells. These studies suggest that EVs secreted from placental cells might have an essential role in maternal-fetal communication during normal and pathological pregnancies.

Longitudinal sampling allowed us to examine canonical pathways represented by PLAP^+^–EV proteome cargo at different stages of gestation, indicative of placental function. Bioinformatic analysis using IPA analysis (see supplemental figure 1) noted differences between pathways represented in placental EVs in both term and preterm pregnancies. First trimester proteome compared with second, third, and term pregnancies revealed changes in acute phase response proteins, ephrin receptor signaling, nitric oxide, macrophage-reactive oxygen species, integrin signaling, and Liver X Receptor/Retinoid X Receptor (LXR/RXR) pathways during normal pregnancy. These pathways collectively represented nonspecific inflammatory response. Nonspecific inflammation was higher in the second trimester than in the first, as well as the third trimester. This is supportive of localized inflammation and a hyperoxic state required for fetoplacental growth during the second trimester. This nonspecific inflammation was minimized in the third trimester but built up at term, likely to facilitate parturition. Interleukin (IL)-8 signaling was also increased at term in placental EVs, indicating chemotaxis in the placenta to promote inflammation leading to parturition (64–66). In preterm placental EVs, as in normal placental EVs, nonspecific inflammation was upregulated across gestation and increased with labor, and with term births, IL-8 signaling was also elevated in preterm pregnancies. This suggests that placental development likely has the same trajectory in term and preterm pregnancies. Additionally, first trimester preterm cargo had more inflammatory markers than with term pregnancies. Inflammatory processes are important for early pregnancy tissue remodeling (67) (eg, coagulation and reactive oxygen species/reactive nitrogen species (ROS/RNS) production in macrophages); however, this seems slightly elevated in the placenta during the early stages of preterm labor. Both inflammation and ROS/RNS are essential for tissue remodeling, but their increased expression may indicate a pathologic mechanism. Thus, these data suggest that the differences in the proteomic content in circulating placental EVs at the first trimester may be used to predict pregnancy outcomes. However, this study did not perform independent quantitation (eg, ELISA or Western blot) or any specific functional analysis of differentially expressed proteins. Therefore, we are avoiding speculative discussion on individual proteins represented as cargo in PLAP^+^ EVs.

At term, an enrichment of genes associated with epithelial mesenchymal transition (EMT) were identified in placental EVs compared with sPTB. Recent findings from our laboratory and others have alluded to the fact that placental membranes, not necessarily placenta, undergo EMT at term (68). EMT results in accumulation of large numbers of mesenchymal cells that are highly vulnerable to oxidative stress and inflammation, causing localized inflammation required to promote parturition (68). To note, this cellular level event was represented only in term but not in PTBs. To support this data, our ongoing studies have shown that fetal tissue EMT is associated with term birth but not PTBs unless preterm labor is preceded by preterm prelabor rupture of the membranes (pPROM). PTB was also devoid of placental coagulopathies or complement activation, conditions often associated with pPROM.

Several lines of evidence in both humans and animals indicate a functional role for EVs in fetomaternal tissues. For example, using a cyclic recombinase reporter construct, we have recently demonstrated that small EVs injected on the maternal side can cross over to the fetal side to produce a functional effect in animal models (18). Small EVs from the fetus, irrespective of the source, can also crossover to the maternal side and can be seen in the maternal circulation. Approximately 35% of total small EVs in maternal circulation are fetal in origin after embryonic day 16 in mouse models (18). Similar numbers were reported by Salomon et al in human pregnancies in the second and third trimesters (39,61). Using in vitro models, fetal EVs are shown to cause proinflammatory marker increases (IL-6, IL-8, and PGE2) in uterine decidual and myometrial cells that mimic parturition-related changes in these tissues (19). Moreover, injection of small EVs from inflammatory cargo-rich embryonic day 18 exosomes caused PTB in animals (17). Interestingly, the levels of PLAP^+^ EVs in amniotic fluid and maternal plasma do not differ in term and preterm parturition; however, their proteome cargo is distinctly different and indicates underlying pathogenic mechanisms. Thus, this evidence is suggestive of a functional role for EVs during pregnancy and parturition. However, this study has limitations. Small sample size did not allow us to perform stratified analysis and risk-based profiling of EV cargo in preterm subjects. For example, we have observed low body mass index in our PTB group, and the exosome cargo analysis suggests impairment of lipid and small molecule metabolism, suggestive of nutritional impact on determining PTB pathways. Although we do not have any functional evidence, it is likely that sociodemographic factors contributing to nutritional deprivation resulting in PTB pathways are reflected in our EV cargo analysis. Although this is a descriptive study, several bioinformatics analyses were performed to identify the function of the changes in the protein profile within exosomes in term and PTB pregnancies. However, further experimental studies are required to determine the function and signaling pathways associated with exosomes. Placental EVs and their cargo proteins are implicated in various pathologic conditions associated with various pregnancy complications resulting in indicated preterm deliveries (26,69). Using maternal plasma samples from a longitudinal cohort, this study identified distinct placental functions at various gestational ages in pregnancies ending with term and spontaneous PTB by analyzing differentially regulated proteins encoded in placental EVs. Placental function is not studied in real time as there are no placental-specific measurable markers in noninvasive samples. Ultrasound-guided approaches can indicate placental health but will not reveal any pathophysiology or mechanistic events. The ability to profile the protein content of placenta-derived EVs represents a real-time snapshot of placental physiology (or pathophysiology) and their normal and or abnormal changes predictive of pregnancy outcomes. Thus, we suggest that early determination of circulating placental vesicles and their protein content might be developed as a biomarker of the functional status of the placenta in minimally invasive maternal blood samples.

## Abbreviations

ABC: ammonium bicarbonate
EMT: epithelial mesenchymal transition
EV: extracellular vesicle
FDR: false discovery rate
GCH: Gurugram Civil Hospital
IL: interleukin
GESA: gene set enrichment analysis
Ig: immunoglobulin
IPA: Ingenuity Pathway Analysis
miRNA: micro RNA
NTA: nanoparticle tracking analysis
PBS: phosphate-buffered saline
PE: preeclampsia
PLAP: placental alkaline phosphatase
PTB: preterm birth
RNA: ribonucleic acid
sPTB: spontaneous PTB
SWATH: sequential windowed acquisition of all theoretical mass spectra

## Notes

***Financial Support:*** This work was supported by the Bill and Melinda Gates Foundation to R.M. (PI). C.S. is supported by the Lions Medical Research Foundation, National Health and Medical Research Council Grant 1114013, and by Fondo Nacional de Desarrollo Cientifico y Tecnologico Grant 1170809. The samples were collected under the The Garbh-Ini Study that has been funded by the Department of Biotechnology (DBT), Govt. of India, augmented by Biotechnology Industry Research Assistance Council–All Children Thriving (BIRAC-ACT), India.

***Author Contributions:*** R.M. conceived the idea, generated grant funds, designed studies, data analysis and interpretation, and drafted the manuscript. C.D. dealt with sample preparation, exosome isolation, and proteomics. P.K. dealt with sample collection and coordination of data and manuscript preparation. S.B. provided administrative support, cohort development, and sample collection. A.L. and D.G. dealt with exosome isolation, proteomics, and data analysis. S.S.M. provided pathway analysis, data interpretation, and manuscript preparation. C.S.G. designed the placental exosome studies, analysis, data interpretation, and manuscript preparation.

## Additional Information

***Disclosure Summary:*** The authors have nothing to disclose.

***Data Availability.*** All data generated or analyzed during this study are included in this published article or in the data repositories listed in References.
